# Circulating free T3 associates longitudinally with cardio-metabolic risk factors in euthyroid children with higher TSH

**DOI:** 10.3389/fendo.2023.1172720

**Published:** 2023-05-17

**Authors:** Gemma Carreras-Badosa, Elsa Puerto-Carranza, Berta Mas-Parés, Ariadna Gómez-Vilarrubla, Helena Cebrià-Fondevila, Ferran Díaz-Roldán, Elena Riera-Pérez, Francis de Zegher, Lourdes Ibañez, Judit Bassols, Abel López-Bermejo

**Affiliations:** ^1^Pediatric Endocrinology Group, Girona Biomedical Research Institute, Girona, Spain; ^2^Pediatrics, Dr. Josep Trueta Hospital, Girona, Spain; ^3^Maternal-Fetal Metabolic Group, Girona Biomedical Research Institute, Girona, Spain; ^4^Pediatrics, Fundació Salut Empordà, Figueres, Spain; ^5^Department of Development and Regeneration, University of Leuven, Leuven, Belgium; ^6^Sant Joan de Déu Children’s Hospital Pediatric Research Institute, University of Barcelona, Barcelona, Spain; ^7^CIBER de Diabetes y Enfermedades Metabólicas Asociadas, Instituto de Salud Carlos III, Madrid, Spain; ^8^Department of Medical Sciences, University of Girona, Girona, Spain

**Keywords:** cardio-metabolic risk, children, thyroid hormone, thyroid hormone (T3), obesity

## Abstract

**Introduction:**

Thyroid hormones play major roles in the regulation of body composition and metabolism, and therefore, the relationship between thyroid hormones and cardio-metabolic risk has been extensively studied in adults. In this study, we aimed to test whether free triiodothyronine (fT3) associates longitudinally with cardio-metabolic risk factors in euthyroid children.

**Methods:**

A prospective study cohort of 599 apparently healthy school-age children were assessed at baseline (mean age 8.1 ± 2.1 years), of whom 270 children were also assessed at follow-up (4 years later). Circulating thyroid-stimulating hormone (TSH), free thyroxine (fT4), and fT3 were measured, and cardio-metabolic risk was assessed by means of body mass index (BMI), waist circumference, visceral fat (by ultrasound), blood pressure, circulating lipids, and homeostasis model assessment of insulin resistance (HOMA-IR) index, both at baseline and at follow-up.

**Results:**

All studied children had normal thyroid function tests. Independent associations between baseline fT3 and both baseline and follow-up BMI, systolic blood pressure, mean arterial blood pressure, triglycerides, and HOMA-IR were found using multivariate regression analysis (adjusting for sex and baseline age and BMI). Analyses of effect sizes showed that for each 1 unit-increase in baseline fT3 (pg/ml), follow-up BMI–standard deviation score (SDS) increased by 0.31 units (z-score) and systolic blood pressure by 6.6 units (mmHg). The observed longitudinal associations were more robust in children belonging to the upper TSH tertile who showed higher TSH levels and were characterized by weighing more and having the highest fT3 levels. In these children, for each 1 unit-increase in baseline fT3 (pg/ml), follow-up BMI-SDS increased by 0.67 units (z-score) and systolic blood pressure by 10.2 units (mmHg).

**Conclusions:**

Circulating fT3 associates longitudinally with cardio-metabolic risk factors in euthyroid children with higher TSH. The observed associations of thyroid hormones in these children could conceivably respond to a homeostatic attempt to reduce their cardio-metabolic risk.

## 1 Introduction

Obesity, and especially childhood obesity, is nowadays a significant worldwide public health problem, as excessive accumulation of body fat leads to an increased risk of developing cardio-metabolic diseases later in life (1).

Thyroid hormones control both body composition and body growth (2) and regulate metabolism as well (3); thus, the interest in the relationship between thyroid hormones and cardio-metabolic risk is on the rise.

Thyroid hormone levels have been extensively studied in relation to dysmetabolic phenotypes in both adults and also as early as in prepubertal children. Initial studies analyzed the associations of circulating thyroid hormones within normal levels with insulin resistance (4), metabolic syndrome (5), and cardio-metabolic risk factors (6) in adults. Circulating T4 was inversely associated with components of the metabolic syndrome in adults (5) and inversely associated with insulin resistance and visceral fat accumulation in children (7). Several epidemiological studies have investigated the association between thyroid-stimulating hormone (TSH) and obesity (8). As opposed to T4, TSH levels have been directly associated with waist circumference in adults (9), and it is known that an increase in body mass index (BMI), progressive central fat accumulation, and/or genetically driven BMI can cause an elevation of serum TSH (10), (11).

Recently, T3 (the active hormone) and T3/T4 ratio (a surrogate marker of the deiodinase activity) have also been studied in relation to cardio-metabolic risk factors, especially in adults in whom they have been related to insulin levels (12) and a less favorable metabolic profile (13). However, studies on the associations between T3 and cardio-metabolic parameters in children are scarce; data on the longitudinal association of thyroid function with cardio-metabolic parameters during childhood are lacking.

In view of this background, we hypothesized that fT3 would be associated with cardio-metabolic risk factors in children. We therefore aimed to study free T3 (fT3) serum levels in relation to cardio-metabolic parameters such as weight, BMI, waist, visceral fat mass, blood pressure, triglycerides, high-density lipoprotein cholesterol (HDL)-cholesterol, and homeostasis model assessment of insulin resistance (HOMA-IR) index in apparently healthy children both at baseline and longitudinally during childhood.

## 2 Material and methods

### 2.1 Study population and ethics

A review of the data on thyroid function in 599 apparently healthy school-age children (291 girls and 308 boys) was performed. Subjects were consecutively recruited among those seen in primary care settings in Girona and Figueres, regions in North-eastern Spain, as previously reported (14). A follow-up visit was offered to all the families after 4 years of the initial baseline visit, and follow-up data were finally available in 270 of the children (141 girls and 129 boys).

Briefly, the inclusion criterion was age between 6 and 10 years. The exclusion criteria were i) major congenital anomalies; ii) abnormal blood count and abnormal liver, kidney, or thyroid functions; iii) evidence of chronic illness or prolonged use of medication; iv) acute illness or use of medication in the month preceding potential enrolment. All children were euthyroid, with TSH and free thyroxine (fT4) values within published reference values (15).

The study protocol was approved by the Ethics Review Committee of the Institutional Review Board of Dr Josep Trueta Hospital and was performed in accordance with their code of ethics, guidelines, and regulations. Informed written consent was obtained from the parents.

### 2.2 Clinical assessments and laboratory variables

A clinical examination followed by venous blood sampling in the fasting state was performed in the morning as previously reported (14).

Briefly, weight and height were measured with a calibrated scale and a Harpenden stadiometer, respectively. BMI was calculated as weight divided by the square of height in meters. Age-adjusted and sex-adjusted standard deviation scores (SDSs) for BMI were calculated using regional normative data (16). Waist circumference was measured in the supine position at the umbilical level, and hip circumference was measured at the widest part, at the level of the greater trochanters, with a metric tape. Blood pressure was measured in the supine position on the right arm after a 10-min rest using an electronic sphygmomanometer with a cuff size appropriate for children’s arm circumference. Mean arterial pressure (MAP) was calculated following this formula: MAP = (systolic blood pressure + (2 * diastolic blood pressure))/3. Puberty was evaluated according to the Tanner criteria.

Visceral fat was estimated by ultrasonography (MyLab 25; Esaote, genoa, Italy) and calculated as described by Hirooka et al. (17) using the distance between the internal surface of the abdominal wall and the posterior wall of the aorta at the umbilical level and the thickness of the fat layer of the posterior right renal wall in the perinephric space. Visceral fat measured by ultrasound has been shown to correlate well with that measured by computed tomography (17). Images were obtained in the supine position at the end of a normal exhalation, using a convex 3.5-MHz transducer. Averages of three measurements of each parameter were used in the study. All measurements were performed by the same observer who was unaware of the clinical and laboratory characteristics of the subjects. Intra-subject coefficient of variation for ultrasound measurements was less than 6%.

All serum samples were obtained between 8:00 and 9:00 AM under fasting conditions. Fasting serum immunoreactive glucose was assayed by the hexokinase method (Cobas C, Roche Diagnostics, Indianapolis, IN, USA), and insulin was measured by immunochemiluminescence (IMMULITE 2000, Diagnostic Products, Los Angeles, CA, USA). The lower detection limit was 2.0 mg/dl and 0.4 mIU/L, and intra- and inter-assay coefficients of variation (CVs) were <3% and <10%, respectively. Insulin resistance was estimated by the homeostasis model assessment index: HOMA-IR = (fasting insulin in mU/L * fasting glucose in mg/dl)/405. Total serum triacylglycerol (triglycerides) was measured by the glycerol-phosphate oxidase method (ARCHITECT, Abbott Laboratories, Abbott Park, IL, USA). The lower detection limit was 5.0 mg/dl, and intra- and inter-assay CVs were <5%. HDL-cholesterol was quantified by the homogeneous method of selective detergent with an accelerator. Serum TSH, fT3, and fT4 were measured by a chemiluminescent microparticle immunoassay (Abbott Laboratories, Abbott Park, IL, USA), with respective detection limits of 0.01 mIU/L, 0.9 pg/ml, and 0.4 ng/dl; coefficients of variation were respectively <5%, <8%, and 8%. The fT3/fT4 ratio is a calculated index used to indicate thyroid function and the action of hormones on the tissue (indirect index of deiodinase activity), which was calculated by dividing the concentration of fT3 by fT4 in conventional units.

Follow-up data were obtained 4 years after the baseline visit, and the same anthropometric, clinical, and laboratory variables were assessed following the same methodology.

### 2.3 Statistics

Statistical analyses were performed using SPSS version 22.0 (SPSS Inc.). Results are expressed as mean ± standard deviation (SD) for the continuous variables or percentage (%) for categorical variables. Logarithmic transformation was used to obtain normally distributed values for triglycerides. Differences across TSH subgroups defined by TSH tertiles were examined by one-way ANOVA test (continuous data) and by chi-square (categorical data). The ANOVA results were corrected for multiple comparisons using the Bonferroni *post-hoc* test. Levels of the studied thyroid hormones were correlated with anthropometric and clinical variables using Pearson’s bivariate correlations. Correlation analyses were followed by multivariate linear regression analyses in which the enter method was used for computing the independent variables and the step-wise method was used for calculating effect sizes (multivariate analyses were adjusted for covariates: sex, baseline age, and baseline BMI). Finally, the interaction of baseline TSH levels (tertiles) in the association between fT3 levels and the studied variables was tested with univariate generalized linear model (GLM). The significance level was set at p ≤ 0.05.

## 3 Results

### 3.1 Children with higher concentrations of TSH and free T3 have a higher cardio-metabolic risk

Body composition and metabolic variables are summarized in **Table 1** for all studied subjects as well as for subgroups according to tertiles of baseline TSH levels.

**Table 1:** Descriptive values of the studied variables in euthyroid children at the baseline visit (6–10 years old) and the follow-up visit, both in all subjects and in subgroups thereof according to baseline TSH tertiles.

	All subjects	Baseline TSH tertiles		
		Lower tertile 0.42–2.13 mUI/L	Medium tertile 2.14–3.11 mUI/L	Upper tertile 3.12–5.94 mUI/L
	Baseline visit (N = 599)	Baseline visit (N = 202)	Baseline visit (N = 198)	Baseline visit (N = 199)
Age (years)	8.1 ± 2.1	7.9 ± 2.0	8.1 ± 2.2	8.4 ± 2.1*
Birth weight (g)	3,228.5 ± 561.4	3,221.2 ± 563.0	3,275.8 ± 522.2	3,188.9 ± 596.2
Birth weight-SDS	0.14 ± 1.40	0.15 ± 1.26	0.21 ± 1.48	0.06 ± 1.46
Sex (female; %)	48%	52.0%	46.9%	47.0%
Puberty (≥Tanner 2 stage; %)	12.0%	9.9%	10.7%	15.0%
Obesity (≥2 BMI-SDS; %)	19.0%	10.0%	18.8%	32.5%^Ø^
Weight (kg)	35.7 ± 16.0	32.7 ± 14.3	35.2 ± 15.7	38.9 ± 17.4***#
Weight-SDS	0.72 ± 1.50	0.46 ± 1.34	0.66 ± 1.42	1.03 ± 1.67***#
Height (cm)	131.5 ± 15.5	129.7 ± 15.4	131.0 ± 15.7	133.5 ± 15.1*#
Height-SDS	0.43 ± 1.27	0.39 ± 1.38	0.39 ± 1.22	0.51 ± 1.21
BMI (kg/m^2^)	19.62 ± 4.81	18.60 ± 4.08	19.44 ± 4.53	20.81 ± 5.48***#Λ
BMI-SDS	0.62 ± 1.42	0.34 ± 1.21	0.57 ± 1.31	0.96 ± 1.65***#Λ
Change birth weight-SDS-to-BMI-SDS	0.50 ± 1.84	0.22 ± 1.57	0.42 ± 1.70	0.85 ± 2.14**#
Waist (cm)	64.5 ± 14.4	61.2 ± 12.6	64.4 ± 13.9	67.7 ± 15.7***#
Hip (cm)	72.5 ± 14.9	69.9 ± 14.1	71.7 ± 14.3	75.7 ± 15.7***#Λ
Visceral fat (mm)	6.52 ± 2.12	6.17 ± 1.89	6.49 ± 2.22	6.84 ± 2.18**#
Systolic blood pressure (mmHg)	105 ± 11	103 ± 11	104 ± 10	108 ± 11***#Λ
Diastolic blood pressure (mmHg)	60 ± 8	59 ± 8	60 ± 8	61 ± 9**#
Mean arterial blood pressure (mmHg)	75 ± 8	73 ± 8	75 ± 7	77 ± 8***#Λ
Triglycerides (mg/dl)	63 ± 30	56 ± 21~	63 ± 28	70 ± 37***#
HDL-cholesterol (mg/dl)	56 ± 12	56 ± 12	57 ± 12	56 ± 13
Glucose (mg/dl)	87 ± 7	86 ± 6	86 ± 6	88 ± 7*
Insulin (μUI/ml)	6.1 ± 5.2	4.8 ± 4.0	6.0 ± 5.1	7.3 ± 6.9**#
HOMA-IR	1.31 ± 1.03	1.04 ± 0.98	1.31 ± 1.08	1.56 ± 1.06***#
TSH (mUI/L)	2.76 ± 1.13	1.60 ± 0.36~	2.61 ± 0.26	4.09 ± 0.72***#Λ
fT3 (pg/ml)	4.11 ± 0.56	3.92 ± 0.46~	4.17 ± 0.59	4.24 ± 0.56***#
fT4 (ng/dl)	1.19 ± 0.15	1.18 ± 0.14	1.20 ± 0.16	1.19 ± 0.14
fT3/fT4 ratio	3.48 ± 0.51	3.36 ± 0.49~	3.51 ± 0.44	3.59 ± 0.55***#
	Follow-up visit (N = 270)	Follow-up visit (N = 94)	Follow-up visit (N = 89)	Follow-up visit (N = 87)
Age (years)	11.8 ± 2.1	11.6 ± 2.2	11.7 ± 2.0	11.9 ± 2.1
Sex (female; %)	52%	55.8%	44.9%	55.1%
Puberty (≥Tanner 2 stage; %)	39.0%	32.6%	36.0%	47.2%
Obesity (≥2 BMI-SDS; %)	13.0%	7.9%	13.5%	17.2%
Weight (kg)	50.1 ± 19.0	48.4 ± 17.9	48.3 ± 17.2	53.4 ± 21.2
Weight-SDS	0.54 ± 1.43	0.41 ± 1.32	0.50 ± 1.42	0.69 ± 1.55
Height (cm)	151.8 ± 14.0	150.3 ± 14.6	151.6 ± 12.6	153.1 ± 14.7
Height-SDS	0.38 ± 1.08	0.31 ± 1.13	0.45 ± 1.04	0.36 ± 1.10
BMI (kg/m^2^)	21.21 ± 5.43	20.76 ± 4.63	20.78 ± 5.15	22.04 ± 6.34
BMI-SDS	0.42 ± 1.43	0.31 ± 1.20	0.33 ± 1.39	0.60 ± 1.67
Change birth weight-SDS-to-BMI-SDS	0.18 ± 1.76	0.11 ± 1.49	0.13 ± 1.51	0.30 ± 2.21
Waist (cm)	73.5 ± 13.7	72.1 ± 11.8	73.1 ± 13.6	75.1 ± 15.4
Hip (cm)	82.1 ± 15.8	79.5 ± 14.9	81.4 ± 14.8	85.0 ± 16.8
Visceral fat (mm)	6.49 ± 1.95	6.34 ± 1.66	6.32 ± 1.86	6.85 ± 2.31
Systolic blood pressure (mmHg)	109 ± 13	107 ± 12	108 ± 13	110 ± 13
Diastolic blood pressure (mmHg)	60 ± 8	59 ± 7	59 ± 7	62 ± 9*#
Mean arterial blood pressure (mmHg)	76 ± 8	75 ± 8	75 ± 8	78 ± 9*#
Triglycerides (mg/dl)	62 ± 27	60 ± 21	64 ± 28	63 ± 30
HDL-cholesterol (mg/dl)	57 ± 13	56 ± 12	57 ± 14	57 ± 13
Glucose (mg/dl)	87 ± 7	87 ± 7	87 ± 7	87 ± 7
Insulin (μUI/ml)	8.1 ± 6.1	7.5 ± 5.2	7.1 ± 4.7	9.9 ± 7.8*#Λ
HOMA-IR	1.72 ± 1.22	1.63 ± 1.17	1.53 ± 1.07	2.01 ± 1.37*Λ
TSH (mUI/L)	2.48 ± 1.21	1.70 ± 0.54~	2.40 ± 0.86	3.37 ± 1.40***#Λ
fT3 (pg/ml)	3.86 ± 0.53	3.71 ± 0.47	3.88 ± 0.52	4.01 ± 0.57***#
fT4 (ng/dl)	1.17 ± 0.13	1.17 ± 0.12	1.15 ± 0.13	1.18 ± 0.13
fT3/fT4 ratio	3.32 ± 0.53	3.19 ± 0.46~	3.38 ± 0.53	3.42 ± 0.55**#

Data are shown as mean ± standard deviation (SD).

Chi-square test: ^Ø^p-value <0.01.

ANOVA test, linear p-value for trend: *p-value <0.05, **p-value <0.01, ***p-value <0.001.

ANOVA Bonferroni post-hoc tests: #p-value <0.05 tertile 1 vs. tertile 3; Λp-value <0.05 tertile 2 vs. tertile 3; ~p-value <0.05 tertile 1 vs. tertile 2.

BMI, body mass index; SDS, standard deviation score; HOMA-IR, homeostasis model assessment index of insulin resistance; TSH, thyroid-stimulating hormone; fT3, free T3; fT4, free T4.

Children in the upper baseline TSH tertile (subgroup composed of those subjects with higher baseline TSH levels) showed higher concentrations of fT3, fT3/fT4 ratio, and TSH (as expected) both at baseline and at follow-up (ANOVA linear p-value for trend <0.001; **Table 1**). This group also depicted a higher percentage of children with obesity according to a cutoff value of BMI-SDS > 2 (chi-square test p-value <0.01; **Table 1**) and higher values for the studied parameters at baseline and at follow-up (ANOVA Bonferroni *post-hoc* test p-value <0.05; **Table 1**). Consequently, the upper TSH tertile group comprised those children with higher thyroid hormone levels and higher values for cardio-metabolic risk factors. This observation set also the basis for further analyses examining the association of fT3 with cardio-metabolic risk in our study.

### 3.2 Free T3 is associated with several cardio-metabolic parameters in healthy euthyroid children

In all studied children, multivariate regression analysis (adjusting for sex, baseline age, and baseline BMI) showed independent positive associations between baseline fT3 and baseline body composition parameters as well as metabolic parameters [correlations shown in **Supplementary Figure S1**, Pearson’s coefficients (all r > 0.245) marked in bold in **Table 2** and beta coefficients shown in **Supplementary Table S1**]. Longitudinally, baseline fT3 is also associated independently with the same parameters at follow-up [correlations shown in **Supplementary Figure S1**, Pearson’s coefficients (all r > 0.211) marked in bold in **Table 2** and beta coefficients shown in **Supplementary Table S1**].

**Table 2:** Correlation coefficients between fT3 serum levels and the studied parameters in euthyroid children at baseline and at follow-up (after 4 years), both in all subjects and in subgroups thereof according to baseline TSH tertiles.

Baseline visit	All subjects	Baseline TSH tertiles		
		Lower tertile 0.42–2.13 mUI/L	Medium tertile 2.14–3.11 mUI/L	Upper tertile 3.12–5.94 mUI/L
	Baseline fT3	Baseline fT3	Baseline fT3	Baseline fT3
	(N = 599)	N = 202	N = 198	N = 199
Change birth weight-SDS-to-BMI-SDS	0.263***	0.130	0.160*	0.386***
Weight-SDS	0.340***	0.279***	0.267***	0.400***
BMI-SDS	0.351***	0.265***	0.274***	0.416***
Waist	0.333***	0.324***	0.244**	0.360***
Systolic blood pressure	0.304***	0.324***	0.189**	0.338***
Diastolic blood pressure	0.315***	0.228**	0.251***	0.407***
Mean arterial blood pressure	0.346***	0.296***	0.253***	0.429***
Triglycerides (log)	0.245***	0.130	0.166*	0.308***
HOMA-IR	0.320***	0.302***	0.243**	0.352***
HOMA-IR-to-HDL ratio (log)	0.336***	0.298***	0.281***	0.376***
	Baseline fT3	Baseline fT3	Baseline fT3	Baseline fT3
Follow-up visit	(N = 270)	N = 94	N = 89	N = 87
Change birth weight-SDS-to-BMI-SDS	0.278***	0.011	0.239*	0.482***
Weight-SDS	0.325***	0.308**	0.206	0.448***
BMI-SDS	0.337***	0.239*	0.223*	0.495***
Waist	0.293***	0.293**	0.191	0.375***
Systolic blood pressure	0.322***	0.345**	0.173	0.440***
Diastolic blood pressure	0.077	0.048	−0.198	0.261*
Mean arterial blood pressure	0.211**	0.208	−0.024	0.378***
Triglycerides (log)	0.183**	0.136	0.001	0.398***
HOMA-IR	0.267***	0.160	0.209	0.380***
HOMA-IR-to-HDL ratio (log)	0.256***	0.129	0.196	0.437***

Pearson’s correlation coefficients (r) are shown; *p-value <0.05, **p-value <0.01, ***p-value <0.001.

Bold type highlights the independent associations after correcting for confounding variables (sex, baseline age, and BMI) in multiple regression analyses.

BMI, body mass index; SDS, standard deviation score; HOMA-IR, homeostasis model assessment of insulin resistance; TSH, thyroid-stimulating hormone; fT3, free T3; fT4, free T4.

The effect sizes of the associations between baseline fT3 and the studied parameters were assessed using multivariate models (unstandardized B values). For each 1 unit-change in baseline fT3 (pg/ml), follow-up BMI-SDS increased by 0.31 units (z-score) and systolic blood pressure by 6.6 units (mmHg). Other effect sizes are shown in the table (**Supplementary Table S2**).

Next, we tested the interaction of baseline TSH levels (tertiles) in the associations between fT3 and the studied cardio-metabolic variables. We observed a significant interaction of “fT3 * baseline TSH tertiles” in the association of baseline fT3 with baseline BW-to-BMI-SDS change, BMI-SDS, systolic blood pressure, and mean arterial blood pressure, as well as with follow-up BW-to-BMI-SDS change, BMI-SDS, diastolic blood pressure, mean arterial blood pressure, and triglycerides (**Supplementary Table S3**). We therefore tested the associations of fT3 with cardiovascular risk factors in subgroups defined by TSH tertiles.

### 3.3 The associations between free T3 and cardio-metabolic parameters were more pronounced in children with higher TSH

At baseline, fT3 is associated with several body composition and metabolic parameters (**Table 2**) across all TSH subgroups.

**Figure 1:**
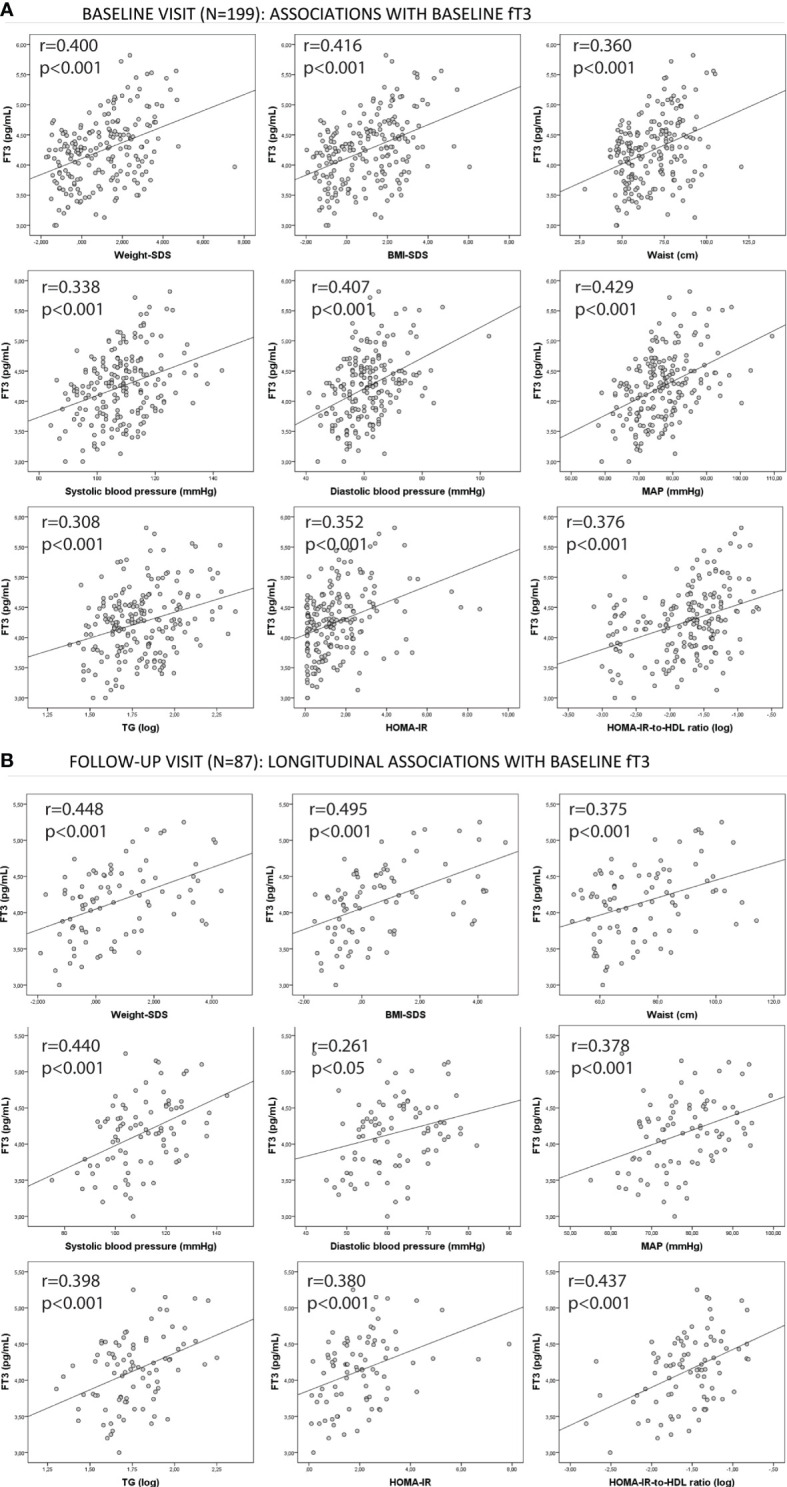
Associations between baseline fT3 and the studied cardio-metabolic parameters both at **(A)** baseline and at **(B)** follow-up in the upper baseline TSH tertile group. Pearson’s correlation coefficients (r) and p-values are shown. BMI, body mass index; MAP, mean arterial blood pressure; HOMA-IR,homeostasis model assessment of insulin resistance; fT3, free T3.

Longitudinally, most of the associations between baseline fT3 and follow-up parameters were more pronounced in the upper TSH tertile subgroup. Of note, multivariate regression analysis (adjusting for sex, baseline age, and baseline BMI) showed independent associations between baseline fT3 and follow-up change BW-to-BMI-SDS, weight-SDS, BMI-SDS, systolic blood pressure, mean arterial blood pressure, triglycerides, HOMA-IR, and HOMA-IR-to-HDL ratio only in the upper TSH tertile (those children with higher baseline TSH values and also at higher cardio-metabolic risk) [correlations shown in **Figure 1**, Pearson’s coefficients (all r > 0.378) marked in bold in **Table 2**, and beta coefficients shown in **Supplementary Table S4**].

Finally, the effect sizes of the associations between baseline fT3 and the studied parameters were also assessed in the upper TSH tertile group. We found that for each 1 unit-change in baseline fT3 (pg/ml), follow-up BMI-SDS increased by 0.67 units (z-score) and systolic blood pressure by 10.2 units (mmHg). Other effect sizes are shown in the table (**Supplementary Table S5**).

## 4 Discussion

First, our results indicate that healthy euthyroid children with higher TSH showed a higher cardio-metabolic risk, as assessed by their higher values of cardiovascular risk factors. Second, baseline fT3 was independently and longitudinally associated with several cardio-metabolic risk parameters in these children, including weight-SDS, BMI-SDS, systolic blood pressure, triglycerides, and HOMA-IR. Third, the associations between baseline fT3 and such cardiovascular risk parameters were more robust in children who presented high baseline TSH levels (upper TSH tertile).

The underlying causal association between TSH and obesity remains unclear (8), although obesity seems to be the cause of the rise in TSH serum levels (10). Some studies have observed a relationship between ectopic liver fat accumulation and TSH levels in adults (18). Others observed that TSH levels could be predictive of weight loss after a dietary intervention in subjects with obesity, with those with higher baseline TSH values showing a better response to the intervention (19). Finally, a progressive accumulation of adiposity was associated with a rise in both TSH and fT3 in adults (9), as well as in children with obesity (11).

Despite knowing that obesity is associated with increased levels of both TSH and T3, thyroid hormone changes can also be an adaptation process aimed at increasing energy expenditure in an attempt to reduce or prevent further weight gain (20) or as an adaptation to fat excess (21). It is known that T3 influences lipid turnover in adipocytes; in turn, TSH controls lipid storage through the regulation of both lipolysis and lipogenesis (22). In this sense, thyroid hormones may induce changes in the basal metabolic rate, which could consequently affect body composition (22). We could then speculate that the rise in TSH and fT3 observed in children at higher cardio-metabolic risk (presenting higher BMI and higher visceral fat) could respond to an adaptive homeostatic mechanism in an attempt to reduce ectopic adiposity and, in turn, to reduce such cardio-metabolic risk.

Circulating thyroid hormones are maintained within a normal range by a feedback mechanism driven by the hypothalamic–pituitary–thyroid axis, and in targeted tissues, the adequate efficacy of thyroid hormones depends on the availability of thyroid hormone transporters, deiodinases, and receptors, among others. A number of authors postulated that the mechanisms underlying the thyroid hormone alterations, such as elevated TSH, are dependent on leptin (20) or thyroid hormone resistance (23), (24). Leptin is secreted from adipose tissue and modulates the hypothalamic–pituitary axis by regulating thyrotropin-releasing hormone (TRH) secretion and, subsequently, can cause changes in TSH and peripheral thyroid hormone levels (20). However, resistance to thyroid hormones is a state in which the responsiveness of target organs to thyroid hormones is reduced. For example, pituitary T3 resistance resulting from an impaired negative feedback mechanism between TSH and the peripheral thyroid hormones (possibly as a result of a reduced number of T3 receptors in the pituitary gland) could also explain the commonly observed raised TSH concentrations in subjects with obesity (11), (23). Apart from pituitary resistance, patients with obesity can develop peripheral resistance to thyroid hormones; indeed, reduced expression of TSH receptors has been found in peripheral tissues such as adipose tissue in patients with obesity (24). A decreased tissue responsiveness to circulating thyroid hormones would also explain the consequent compensatory increased secretion of TSH and T3 (24). Studies have also shown that higher muscle mass in subjects with obesity relates to an increased fT3/fT4 ratio, possibly reflecting the action of deiodinase activity in skeletal muscle (25). Finally, a higher deiodinase activity in adipose tissue could also contribute to increased T3 levels, as the activity of deiodinase in fat tissue is raised by increased body weight and leptin action (26), (27).

Despite the well-recognized effect of the thyroid hormones (including fT3) on cardiovascular and/or obesity parameters in adults (28), (29). the relationship between fT3 levels and cardio-metabolic risk remains unclear in children. We hypothesized and disclosed that fT3 levels were associated longitudinally with several cardio-metabolic risk factors. Our observations are in line with those reported by other authors in different populations. For example, recent studies also found that fT3 is associated with central adiposity in young adults (30) and with BMI in adolescents (31). Despite the well-known cardiovascular effects of thyroid hormones, few studies have systematically assessed the relationship between thyroid hormones and blood pressure. Only recently, a study demonstrated that fT3 was positively related to the prevalence of elevated blood pressure in euthyroid adults (32). Moreover, we observed a correlation between fT3 and metabolic parameters such as triglyceride levels and the HOMA-IR index. Available data to date are conflicting to what extent thyroid dysfunction is associated with insulin resistance (IR). Some authors aimed to identify which thyroid parameters are predictors of IR, and results showed that, in adults, the strongest identified positive correlations were those between fT3 and insulin levels (12), (33). Correlations between TSH and cholesterol, triglycerides, insulin, and HOMA-IR were previously observed in a cohort of children with obesity (34) as well as in a longitudinal study of children with obesity showing a high-normal range of TSH levels (35).

We would like to point out some of the strengths and limitations of our study. The strengths of the present study are the assessment of thyroid data in children as early as at school age and also the longitudinal design of our study. Listing the limitations, the iodine status of the children was not available, although a study performed in the same region concluded that the iodine status of the population in the studied region is globally acceptable (36). Second, even taking into account the longitudinal design of our study, further experimental studies should be performed to confirm our hypothesis of the activation of the thyroid system in children at higher cardio-metabolic risk. Third, measurements of leptin levels of the studied children were not available. Finally, data on thyroid size or anti-thyroid antibodies were not available. Despite this limitation, we do not expect our cohort to have included cases of subclinical hypothyroidism or autoimmune thyroiditis, as our studied population had normal values for thyroid function tests.

In conclusion, circulating concentrations of free T3 are associated longitudinally with cardio-metabolic risk factors in apparently healthy euthyroid children with higher TSH. The observed associations of thyroid hormones in these children could conceivably respond to a homeostatic attempt to reduce their cardio-metabolic risk.

## Data availability statement

The raw data supporting the conclusions of this article will be made available by the authors, without undue reservation.

## Ethics statement

The studies involving human participants were reviewed and approved by Ethics Review Committee of the Institutional Review Board of Dr Josep Trueta Hospital. Written informed consent to participate in this study was provided by the participants’ legal guardian/next of kin.

## Author contributions

GC-B: Conceptualization, Formal analysis, Visualization and Writing - original draft preparation; EP-C: Conceptualization and Formal analysis; BM-P, AG-V, HC-F, FD-R, and ER-P: Formal analysis; FZ, LI, and JB: Writing – reviewing and editing; and AL-B: Conceptualization, Supervision and Writing – reviewing and editing. All authors contributed to the article and approved the submitted version.
