# Supplementary material for: Circulating free T3 associates longitudinally with cardio-metabolic risk factors in euthyroid children with higher TSH
Source: Front Endocrinol (Lausanne). 2023 May 17;14:1172720. doi: 10.3389/fendo.2023.1172720 (PMC10230068; doi:10.3389/fendo.2023.1172720)
Supplement: Supplementary file 1 [file DataSheet_1.pdf]

Circulating free T3 associates longitudinally with cardio-metabolic risk factors in euthyroid children with higher TSH

Gemma Carreras-Badosa[1*], PhD, Elsa Puerto-Carranza[1,2*], MD, Berta Mas-Parés[1], MSc, Ariadna Gómez-Vilarrubla[3], MSc, Helena Cebrià Fondevila[2], MD, Ferran Díaz-Roldán[1], MSc, Elena Riera-Pérez[4], MD, Francis de Zegher[5], Prof., Lourdes Ibañez[6, 7], Prof., Judit Bassols[3#], PhD, Abel López-Bermejo[1, 2, 8#], PhD.

[1] Pediatric Endocrinology Group, Girona Biomedical Research Institute, Girona, Spain;

[2] Pediatrics, Dr. JosepTrueta Hospital, Girona, Spain;

[3] Maternal-Fetal Metabolic Group, Girona Biomedical Research Institute, Girona, Spain;

[4] Pediatrics, Fundació Salut Empordà, Figueres, Spain;

[5] Department of Development & Regeneration, University of Leuven, Leuven, Belgium;

[6] Sant Joan de DéuChildren's Hospital Pediatric Research Institute, University of Barcelona, Barcelona, Spain;

[7] CIBER de Diabetes y Enfermedades Metabólicas Asociadas, Instituto de Salud Carlos III, Madrid, Spain;

[8] Department of Medical Sciences, University of Girona, Girona, Spain.

***Shared co-first authorships**

***Shared co-senior and co-corresponding authorships**

CORRESPONDING AUTHOR

Abel López-Bermejo, MD

Girona Institute for Biomedical Research

Av. França s/n

Girona-17007. Spain

Tel: +34-972-940200. Ext. 2810

Fax: +34-972-940270

Email: alopezbermejo@idibgi.org

SUPPLEMENTARY DATA

1. **SUPPLEMENTARY TABLES:**

Supplementary Table S1: Multiple regression standardized values (β) and p values of the studied correlations performed in all population (baseline N=599 and follow-up N=270):

**Independent variable: Baseline fT3**				
**Dependent variables used in the multivariate regression analyses:**	**Baseline dependent variables**		**Follow-up dependent variables**	
	β value	*P value*	*β value*	*P value*
Change Birth Weight-SDS-to-BMI-SDS	0.254	< 0.0001	0.130	0.024
Adjusted R Squared		*0.075*		*0.277*
Weight-SDS	0.314	< 0.0001	0.079	0.033
Adjusted R Squared		*0.167*		*0.685*
BMI-SDS	0.323	< 0.0001	0.076	0.026
Adjusted R Squared		*0.179*		*0.731*
Waist	0.032	0.021	0.068	0.058
Adjusted R Squared		*0.899*		*0.707*
Systolic Blood Pressure	0.159	< 0.0001	0.251	< 0.0001
Adjusted R Squared		*0.336*		*0.424*
Diastolic Blood Pressure	0.210	< 0.0001	0.033	0.601
Adjusted R Squared		*0.209*		*0.096*
Mean Arterial Blood Pressure	0.211	< 0.0001	0.148	0.010
Adjusted R Squared		*0.311*		*0.268*
Triglycerides (log)	0.108	0.006	0.109	0.05
Adjusted R Squared		*0.226*		*0.145*
HOMA-IR	0.130	< 0.0001	0.162	0.001
Adjusted R Squared		*0.516*		*0.399*
HOMA-IR-to-HDL ratio (log)	0.147	< 0.0001	0.170	0.001
Adjusted R Squared		*0.501*		*0.348*

Multiple regression standardized coefficients (β), p values and adjusted model R squared values using enter method are shown.

Confounding variables used in the analyses: sex, baseline age and baseline BMI.

BMI: body mass index, SDS: standard deviation score, HOMA-IR: homeostasis model assessment of insulin resistance, TSH: thyroid-stimulating hormone, fT3: free T3.

Supplementary Table S2: Effect size (multiple regression unstandardized values (B), SE values and p values) of the studied associations performed in all population (baseline N=599, follow-up N=270).

**Independent variable: Baseline fT3 (pg/ml)**						
**Dependent variables: increase units**	**Baseline dependent variables**			**Follow-up dependent variables**		
	B value	*SE value*	*P value*	*B value*	*SE value*	*P value*
Change Birth Weight-SDS-to-BMI-SDS (z-score)	0.246	0.121	0.042	0.546	0.207	0.009
Weight-SDS (z-score)	0.143	0.061	0.019	0.283	0.108	0.009
BMI-SDS (z-score)	0.898	0.098	<0.001	0.305	0.103	0.003
Waist (cm)	0.819	0.365	0.025	-	-	*
Systolic Blood Pressure (mmHg)	3.133	0.707	<0.001	6.611	1.323	<0.001
Diastolic Blood Pressure (mmHg)	3.177	0.593	<0.001	-	-	*
Mean Arterial Blood Pressure (mmHg)	3.194	0.549	<0.001	2.548	0.986	0.010
Triglycerides (mg/dl)	5.694	2.037	0.005	-	-	*
HOMA-IR (unit)	0.297	0.072	<0.001	0.400	0.124	0.001
HOMA-IR-to-HDL ratio (unit)	0.141	0.031	<0.001	0.152	0.047	0.001

Multiple regression unstandardized coefficients (B) and SE values using step-wise method are shown.

Confounding variables used in the analyses: sex, baseline age and baseline BMI.

BMI: body mass index, SDS: standard deviation score, HOMA-IR: homeostasis model assessment of insulin resistance, TSH: thyroid-stimulating hormone, fT3: free T3.

* non-significant, fT3 is excluded from the step-wise model

Supplementary Table S3: Univariate General Linear Model (GLM) testing the interaction of baseline TSH tertiles categories in the associations between baseline fT3 with the studied variables at baseline (N=599) and follow-up (N=270).

Dependent variables at baseline	F	Sig.	Dependent variables at follow-up	F	Sig.
**Change Birth Weight-SDS-to-BMI-SDS**			**Change Birth Weight-SDS-to-BMI-SDS**		
Baseline fT3	**31.177**	**<0.0001**	Baseline fT3	**19.371**	**<0.0001**
Baseline TSH (tertiles)	**5.320**	**0.005**	Baseline TSH (tertiles)	**7.987**	**<0.0001**
Baseline TSH (tertiles) * fT3	**6.476**	**0.002**	Baseline TSH (tertiles) * fT3	**8.000**	**<0.0001**
Adjusted R Squared = 0.092			*Adjusted R Squared = 0.118*		
**Weight-SDS**			**Weight-SDS**		
Baseline fT3	**65.860**	**<0.0001**	Baseline fT3	**30.439**	**<0.0001**
Baseline TSH (tertiles)	2.028	0.132	Baseline TSH (tertiles)	1.935	0.147
Baseline TSH (tertiles) * fT3	***2.756***	***0.064***	Baseline TSH (tertiles) * fT3	2.078	0.127
Adjusted R Squared = 0.126			*Adjusted R Squared = 0.107*		
**BMI-SDS**			**BMI-SDS**		
Baseline fT3	**67.955**	**<0.0001**	Baseline fT3	**31.571**	**<0.0001**
Baseline TSH (tertiles)	**3.090**	**0.046**	Baseline TSH (tertiles)	**3.822**	**0.023**
Baseline TSH (tertiles) * fT3	**4.143**	**0.016**	Baseline TSH (tertiles) * fT3	**4.095**	**0.018**
Adjusted R Squared = 0.140			*Adjusted R Squared = 0.129*		
**Waist (cm)**			**Waist (cm)**		
Baseline fT3	**62.180**	**<0.0001**	Baseline fT3	**23.057**	**<0.0001**
Baseline TSH (tertiles)	1.610	0.201	Baseline TSH (tertiles)	1.256	0.287
Baseline TSH (tertiles) * fT3	1.967	0.141	Baseline TSH (tertiles) * fT3	1.405	0.247
Adjusted R Squared = 0.124			*Adjusted R Squared = 0.082*		
**Systolic blood pressure (mmHg)**			**Systolic blood pressure (mmHg)**		
Baseline fT3	**53.987**	**<0.0001**	Baseline fT3	**29.380**	**<0.0001**
Baseline TSH (tertiles)	***2.729***	***0.066***	Baseline TSH (tertiles)	2.022	0.135
Baseline TSH (tertiles) * fT3	**3.165**	**0.043**	Baseline TSH (tertiles) * fT3	2.222	0.110
Adjusted R Squared = 0.124			*Adjusted R Squared = 0.105*		
**Diastolic blood pressure (mmHg)**			**Diastolic blood pressure (mmHg)**		
Baseline fT3	**56.320**	**<0.0001**	Baseline fT3	.805	0.370
Baseline TSH (tertiles)	***2.527***	***0.081***	Baseline TSH (tertiles)	**4.177**	**0.016**
Baseline TSH (tertiles) * fT3	***2.881***	***0.057***	Baseline TSH (tertiles) * fT3	**4.959**	**0.008**
Adjusted R Squared = 0.104			*Adjusted R Squared = 0.046*		
**Mean Arterial blood pressure (mmHg)**			**Mean Arterial blood pressure (mmHg)**		
Baseline fT3	**70.596**	**<0.0001**	Baseline fT3	**10.643**	**0.001**
Baseline TSH (tertiles)	***2.364***	***0.095***	Baseline TSH (tertiles)	**3.918**	**0.021**
Baseline TSH (tertiles) * fT3	**3.024**	**0.049**	Baseline TSH (tertiles) * fT3	**4.502**	**0.012**
Adjusted R Squared = 0.134			*Adjusted R Squared = 0.071*		
**Triglycerides (log)**			**Triglycerides (log)**		
Baseline fT3	**25.633**	**<0.0001**	Baseline fT3	**10.055**	**0.002**
Baseline TSH (tertiles)	2.055	0.129	Baseline TSH (tertiles)	**5.176**	**0.006**

Baseline TSH (tertiles) * fT3	***2.858***	***0.058***	Baseline TSH (tertiles) * fT3	**4.958**	**0.008**
Adjusted R Squared = 0.077			*Adjusted R Squared = 0.053*		
**HOMA-IR**			**HOMA-IR**		
Baseline fT3	**50.954**	**<0.0001**	Baseline fT3	**18.007**	**<0.0001**
Baseline TSH (tertiles)	1.350	0.260	Baseline TSH (tertiles)	1.471	0.232
Baseline TSH (tertiles) * fT3	1.835	0.161	Baseline TSH (tertiles) * fT3	1.948	0.145
Adjusted R Squared = 0.111			*Adjusted R Squared = 0.088*		
**HOMA-IR-to-HDL ratio (log)**			**HOMA-IR-to-HDL ratio (log)**		
Baseline fT3	**60.974**	**<0.0001**	Baseline fT3	**16.518**	**<0.0001**
Baseline TSH (tertiles)	1.060	0.347	Baseline TSH (tertiles)	1.363	0.258
Baseline TSH (tertiles) * fT3	1.237	0.291	Baseline TSH (tertiles) * fT3	1.667	0.191
Adjusted R Squared = 0.114			*Adjusted R Squared = 0.075*		

Univariate GLM testing for main effects (single variables) and interaction. Significant p values <0.05 in bold, and significant p values < 0.10 in italic bold.

BMI: body mass index, SDS: standard deviation score, HOMA-IR: homeostasis model assessment of insulin resistance, TSH: thyroid-stimulating hormone, fT3: free T3.

Supplementary Table S4: Multiple regression standardized values (β) and p values of the studied correlations performed in the upper tertile baseline TSH group (baseline N=199, follow-up N=87).

**Independent variable: baseline fT3**				
**Dependent variables used in the multivariate regression analyses:**	**Baseline dependent variables**		**Follow-up dependent variables**	
	β value	*P value*	*β value*	*P value*
Change BW-to-BMI-SDS	0.381	< 0.0001	0.295	0.004
Adjusted R Squared		*0.143*		*0.370*
Weight-SDS	0.386	< 0.0001	0.144	0.026
Adjusted R Squared		*0.191*		*0.710*
BMI-SDS	0.400	< 0.0001	0.141	0.019
Adjusted R Squared		*0.211*		*0.771*
Waist	0.024	0.253	0.012	0.900
Adjusted R Squared		*0.928*		*0.547*
Systolic Blood Pressure	0.198	0.002	0.370	< 0.0001
Adjusted R Squared		*0.348*		*0.413*
Diastolic Blood Pressure	0.280	< 0.0001	0.200	0.093
Adjusted R Squared		*0.282*		*0.084*
Mean Arterial Blood Pressure	0.280	< 0.0001	0.305	0.005
Adjusted R Squared		*0.381*		*0.246*
Triglycerides	0.103	0.101	0.225	0.028
Adjusted R Squared		*0.362*		*0.304*
HOMA-IR	0.159	0.007	0.203	0.024
Adjusted R Squared		*0.477*		*0.475*
HOMA-IR-to-HDL ratio (log)	0.170	0.003	0.286	0.001
Adjusted R Squared		*0.507*		*0.537*

Multiple regression standardized coefficients (β), p values and adjusted model R squared values using enter method are shown.

Confounding variables used in the analyses: sex, baseline age and baseline BMI.

BMI: body mass index, SDS: standard deviation score, HOMA-IR: homeostasis model assessment of insulin resistance, TSH: thyroid-stimulating hormone, fT3: free T3.

Supplementary Table S5: Effect size (multiple regression unstandardized values (B), SE values and p values) of the studied correlations performed in the upper TSH group (baseline N=199, follow-up N=87).

Independent variable: fT3 (pg/ml)						
**Dependent variables: increase units**	**Baseline dependent variables**			**Follow-up dependent variables**		
	B value	*SE value*	*P value*	*B value*	*SE value*	*P value*
Change Birth Weight-SDS-to-BMI-SDS (z-score)	0.671	0.226	0.003	1.572	0.459	0.001
Weight-SDS (z-score)	0.250	0.112	0.027	0.451	0.198	0.026
BMI-SDS (z-score)	1.241	0.194	<0.001	0.667	0.207	0.022
Waist (cm)	-	-	*	-	-	*
Systolic Blood Pressure (mmHg)	3.794	1.197	0.002	10.151	2.512	<0.001
Diastolic Blood Pressure (mmHg)	4.367	1.057	<0.001	4.645	1.872	0.015
Mean Arterial Blood Pressure (mmHg)	4.317	0.946	<0.001	7.347	1.783	<0.001
Triglycerides (mg/dl)	-	-	*	16.267	6.239	0.011
HOMA-IR (unit)	0.427	0.153	0.006	0.566	0.242	0.024
HOMA-IR-to-HDL ratio (unit)	0.175	0.057	0.003	0.239	0.069	0.001

Multiple Regression unstandardized coefficients (B) and SE values using step-wise method are shown.

Confounding variables used in the analyses: sex, baseline age and baseline BMI.

BMI: body mass index, SDS: standard deviation score, HOMA-IR: homeostasis model assessment of insulin resistance, TSH: thyroid-stimulating hormone, fT3: free T3.

*non-significant, fT3 is excluded from the step-wise model

2. SUPPLEMENTARY FIGURE:

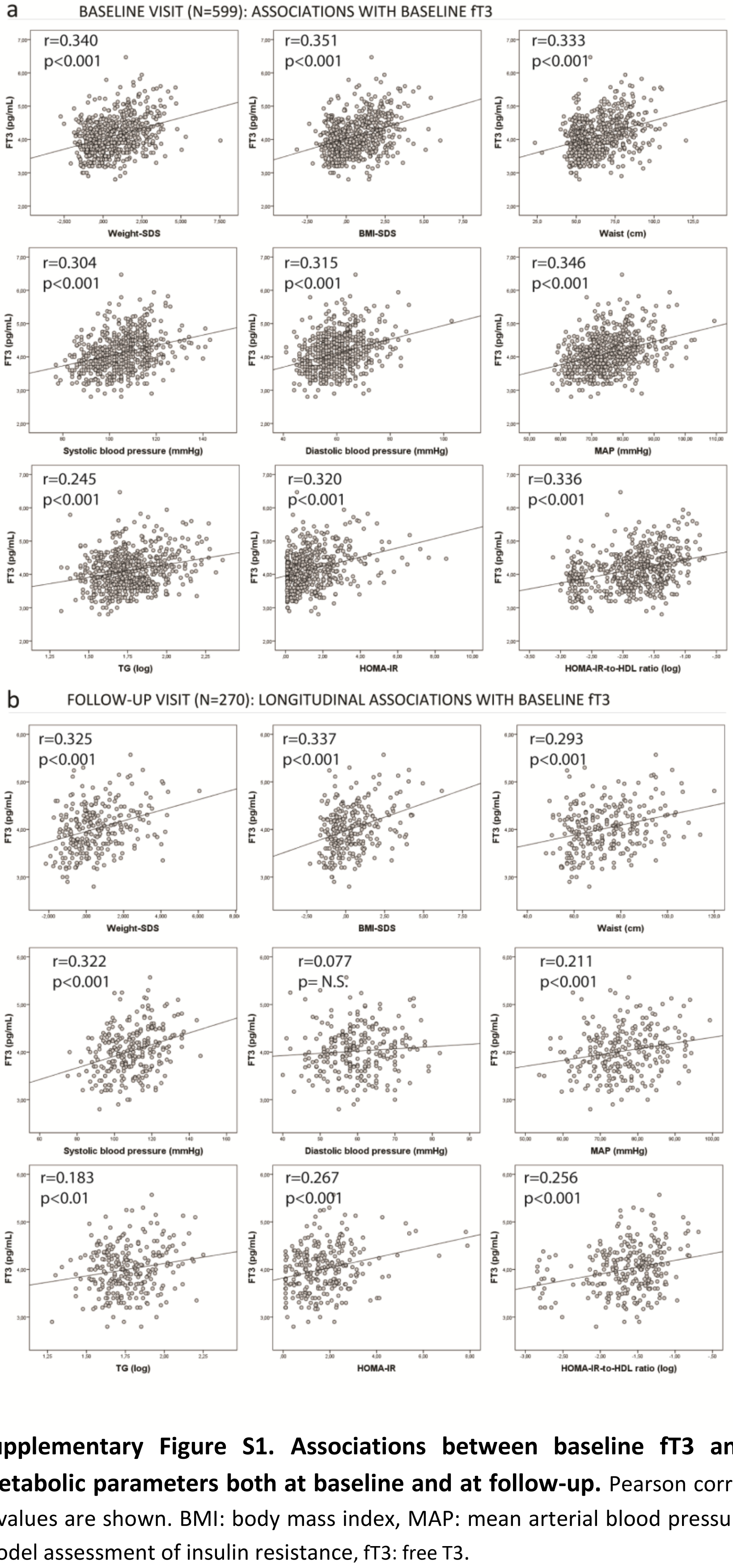

Supplementary Figure S1. Associations between baseline fT3 and the studied cardio-metabolic parameters both at baseline and at follow-up. Pearson correlation coefficients (r) and p-values are shown. BMI: body mass index, MAP: mean arterial blood pressure, HOMA-IR: homeostasis model assessment of insulin resistance, fT3: free T3.
